# Transarterial Chemoembolization Combined With Apatinib Plus PD-1 Inhibitors for Hepatocellular Carcinoma With Portal Vein Tumor Thrombus: A Multicenter Retrospective Study

**DOI:** 10.14309/ctg.0000000000000581

**Published:** 2023-03-15

**Authors:** Wei-Li Xia, Xiao-Hui Zhao, Yuan Guo, Hong-Tao Hu, Guang-Shao Cao, Zhen Li, Wei-Jun Fan, Shi-Jun Xu, Hai-Liang Li

**Affiliations:** 1 Department of Minimal-Invasive Intervention, The Affiliated Cancer Hospital of Zhengzhou University & Henan Cancer Hospital, Zhengzhou, China;; 2 Department of Intervention, Henan Provincial People's Hospital, Zhengzhou, China;; 3 Department of Interventional Radiology, The First Affiliated Hospital of Zhengzhou University, Zhengzhou, China;; 4 Department of Minimally Invasive Interventional Radiology, Sun Yat-sen University Cancer Center, Guangzhou, China.

**Keywords:** hepatocellular carcinoma, portal vein tumor thrombus, transarterial chemoembolization, PD-1 inhibitors

## Abstract

The aim of this study was to compare transarterial chemoembolization (TACE) combined with apatinib and PD-1 inhibitors (TACE-AP) with TACE combined with apatinib alone (TACE-A) in the treatment of hepatocellular carcinoma (HCC) with portal vein tumor thrombus (PVTT) and to explore the prognostic factors affecting the survival of patients.

**METHODS:**

This retrospective study analyzed data of patients with HCC with PVTT who were treated with TACE-AP or TACE-A between December 2018 and June 2021. The primary end points of the study were progression-free survival (PFS) and overall survival (OS), and the secondary end points were objective response rate (ORR) and adverse events (AEs). Propensity score matching (PSM) and stabilized inverse probability weighting (sIPTW) analyses were used to reduce patient selection bias, and Cox regression analysis was used to analyze prognostic factors affecting patient survival.

**RESULTS:**

Sixty-nine and 40 patients were included in the TACE-A and TACE-AP groups, respectively. After PSM and IPTW analyses, the median PFS and median OS in the TACE-AP group were significantly higher than those in the TACE-A group (PFS: after PSM, 6.9 vs 4.0 months, *P* < 0.001, after IPTW, 6.5 vs 5.1 months, *P* < 0.001; OS: after PSM, 14.6 vs 8.5 months *P* < 0.001, after IPTW, 16.1 vs 10.5 months, *P* < 0.001). After PSM and IPTW analyses, the tumor ORR in the TACE-AP group was significantly higher than that in the TACE-A group (PSM, 53.6% vs 17.9%, *P* = 0.005; IPTW, 52.5% vs 28.6%, *P* = 0.013). All treatment-related AEs were observed to be tolerated. Multivariate Cox regression analysis showed that the main prognostic factors affecting the survival of patients were tumor number, PVTT type, alpha-fetoprotein, and treatment mode.

**DISCUSSION:**

In the treatment of patients with HCC with PVTT, TACE-AP significantly improved PFS, OS, and ORR, and the AEs were safe and controllable.

## INTRODUCTION

Primary liver cancer is one of the most common digestive tract malignant tumors and the sixth most common malignant tumor in the world. More than 50% of new cases with liver cancer and deaths occur in China every year, with hepatocellular carcinoma (HCC) as the most common pathological type (1). HCC easily invades the intrahepatic vasculature, especially the portal venous system, and forms portal vein tumor thrombus (PVTT) with an incidence rate of 44.0%–62.2%. The prognosis of these patients is very poor, and the survival period without treatment is only 2–4 months (2). Transarterial chemoembolization (TACE) is an important treatment option for HCC with PVTT in the Asia-Pacific region. Studies have shown that the PVTT feeding artery is a branch of the hepatic artery, which is also the theoretical basis for TACE to have a certain therapeutic effect on PVTT (3).

Apatinib is a tyrosine kinase inhibitor, which acts on the vascular endothelial growth factor (VEGF) and VEGF receptor 2 (VEGFR-2) signaling pathways, inhibits the binding of VEGFR-2 to tyrosine kinase adenosine triphosphate, blocks the proliferation and migration of vascular endothelial cells, and ultimately prevents tumor blood vessels regeneration and reduces tumor recurrence. Previous studies have shown that apatinib combined with TACE has a significant effect in the treatment of advanced HCC. The combination of the 2 inhibits tumor revascularization and reduces tumor volume, ultimately prolonging the survival of patients (4). Immune checkpoint inhibitors act on the body's immune system, effectively block the immune escape and tolerance pathways of tumor cells, and play a role in tumor inhibition through the body's cellular immune function (5). They have been proved to be effective in the treatment of various cancers including liver cancer. Studies have shown that the specific antitumor effects of PD-1 inhibitors and tyrosine kinase inhibitors (TKIs) have theoretical synergistic effects (6). As the basic mode of local treatment of advanced HCC, TACE therapy has been proved to have a positive regulatory effect on the immune level of the body (7). Therefore, it is worth further exploring whether these methods can be comprehensively applied to the treatment of HCC complicated with PVTT to achieve greater survival benefit.

At present, the treatment of HCC complicated with PVTT emphasizes comprehensive treatment to prolong survival and improve quality of life. In the treatment of advanced HCC, the efficacy of TACE combined with apatinib and PD-1 inhibitors (TACE-AP) is very significant (8); however, a controlled study on TACE-AP has not been reported for patients with HCC with PVTT. In this multicenter retrospective study, we explored the efficacy and safety of TACE-AP in the treatment of patients with HCC with portal vein tumor thrombus and analyzed the prognostic factors affecting patient survival.

## METHODS

### Patients

The clinical records of patients with HCC with PVTT who received TACE-A or TACE-AP from December 2018 to December 2021 were retrospectively analyzed. Patients were recruited from the following 3 centers: The Affiliated Cancer Hospital of Zhengzhou University, The First Affiliated Hospital of Zhengzhou University, and Henan Provincial People's Hospital. The Ethics Committee of the Affiliated Cancer Hospital of Zhengzhou University approved this study (approval number: 2017003). The Ethics Committee of other hospitals were informed and agreed to the study. Because of the retrospective design of study, the requirement for informed consent was waived by the Ethics Committee. All extracted data were anonymously analyzed.

**Figure 1.**
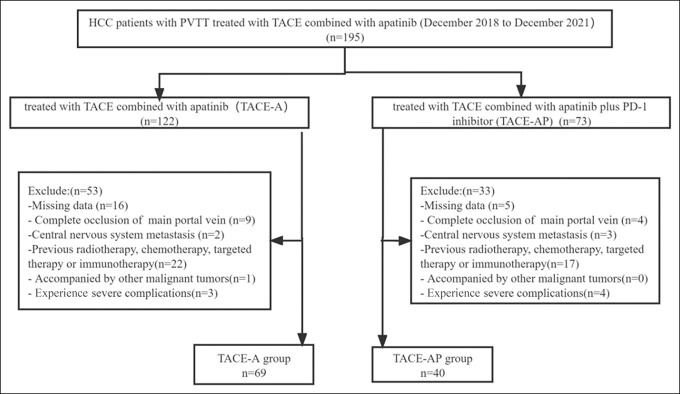
Flow diagram of patient screening. HCC, hepatocellular carcinoma; PVTT, portal vein tumor thrombus; TACE, transarterial chemoembolization.

The following inclusion criteria were applied: (i) clinically or pathologically diagnosed as HCC; (ii) the Barcelona Clinic Liver Cancer stage C with portal vein tumor thrombus; (iii) Child-Pugh class A or B ≤7; (iv) fewer than 10 lesions in the liver and less than 15 cm in any dimension and lesions less than 50% of the volume of the liver; and (v) Eastern Cooperative Oncology Group performance status <2. The exclusion criteria were as follows: (i) complete occlusion of the main portal vein without collateral circulation; (ii) tumor thrombus invasion of the superior mesenteric vein; (iii) metastases invasion of the central nervous system (brain or spinal cord); (iv) received previous systemic therapy, including chemotherapy, targeted therapy, or immunotherapy; (v) concomitant with other malignant tumors; and (vi) experiencing other uncontrollable underlying diseases (including but not limited to hypertension, diabetes, heart disease, etc.) (Figure 1).

Diagnostic criteria for HCC with PVTT consisted of a clear diagnosis of HCC and the presence of typical imaging signs of PVTT (real space-occupying lesions in the portal vein on computed tomography or magnetic resonance imaging (MRI)–enhanced scanning, enhancement in the arterial phase, and filling defect in the portal venous phase). PVTT classification adopts Cheng PVTT classification system proposed by Chinese professor Cheng Shuqun: type I, tumor thrombi involving segmental branches of the portal vein; type II, tumor thrombi involving the right/left portal vein; type III, tumor thrombi involving the main portal vein and trunk; and type IV, tumor thrombus invades superior mesenteric vein (9).

### TACE procedure

The TACE procedure is consistent across centers, and as described in our previous report on the TACE procedure (10), all TACE procedures were performed by 2 experienced minimally invasive interventionalists under local anesthesia through a traditional femoral approach. After routine angiography using a 5F RH catheter (Terumo, Tokyo, Japan), superselective arterial cannulation with a microcatheter (Terumo) was used to access the branch of the feeding artery to the tumor. Doxorubicin (Haizheng Pharmaceutical, Taizhou, China) and lipiodol (Laboratoire Guerbet, Paris, France) were thoroughly mixed and injected into the tumor-nourishing blood vessels and then 560–710 μm gelatin sponge particles (ALICON Pharmaceutical, Hangzhou, China) were administered until blood flow almost stopped. The dosage of lipiodol was 5–20 mL, and the dosage of doxorubicin was 50–70 mg. The actual dose was based on the patient's liver function status, tumor burden, and the patient's body surface area.

### Dosing regimen of apatinib and PD-1 inhibitors

Patients in both TACE-A and TACE-AP groups started oral apatinib (Hengrui Pharmaceutical, Lianyungang, China) 3 days after TACE at a dose of 250 mg/d. Simultaneously, patients in the TACE-AP group received an additional intravenous injection of PD-1 inhibitors (200 mg) every 3 weeks. Dosage adjustment or discontinuation was performed when patients experience serious adverse events (AEs) (≥grade 3). If the AE was judged to be related to apatinib, the apatinib was adjusted to be taken every other day. If the AEs persisted after dose adjustment or AE, namely gastrointestinal bleeding, related to any drug occurred, apatinib administration was temporarily discontinued. When AEs resolved, the dose was restored to 250 mg/d. PD-1 inhibitors were also discontinued if AEs were associated with them, and the drug was resumed after the AEs were eliminated.

### Follow-up and study objectives

Patient follow-up should be performed every 4–6 weeks after TACE. The follow-up included chest computed tomography, liver multiphase-enhanced MRI, routine blood tests, and liver and renal function tests. When MRI results show recurrence or residual activity of the intrahepatic tumor, TACE should be repeated if the patient has good liver function test results (Child-Pugh class A or B ≤7). In this study, patients received apatinib continuously before TACE was repeated, and the treatment was interrupted for 3 days thereafter.

If the patient’s disease progressed during treatment or the treatment was terminated because of intolerable drug toxicity, the treatment plan was changed according to multidisciplinary consultation and the patient's wishes. The follow-up treatment plan was as follows: add PD-1 inhibitors (for patients in the TACE-A group) and use second-line targeted drug regorafenib, radiotherapy, hepatic arterial infusion chemotherapy, or best supportive care. Progression-free survival (PFS) and overall survival (OS) were the primary end points of this study, and prognostic factors affecting survival, objective response rate (ORR), and treatment-related AEs were the secondary end points of this study.

### Evaluation criteria

Patients' progression-free survival was defined as the time from diagnosis to the assessment of progression, and overall survival was defined as the time from diagnosis to death or last follow-up. The evaluation criteria were modified Response Evaluation Criteria in Solid Tumors, and the evaluation results were divided into complete response, partial response, stable disease, and progressive disease. AEs were evaluated according to the National Cancer Institute Common Terminology Criteria for Adverse Events Version 5.0.

### Statistical analysis

To reduce patient selection bias and balance the variables between patients in the 2 groups, we used propensity score matching (PSM) and inverse probability weighting (IPTW) for analysis. A 1:1 ratio was used for PSM analysis with a caliper value of 0.1. Because the weighted sample size is often larger than the original sample size, the increase of the sample size can easily lead to the appearance of false positives. Therefore, the use of stabilized IPTW (sIPTW) can reduce the probability of false-positive events.

Categorical variables were calculated using the χ^2^ test and expressed as percentages, and continuous variables were calculated using the *t* test and expressed as mean ± SD. The median OS and PFS between the 2 groups were estimated using the Kaplan-Meier method. Univariate analysis was used to assess the statistical significance of clinical characteristics, and multivariate Cox regression models were used to include statistically significant variables in the analysis to identify predictors associated with OS. Three cohorts were considered for all analyses: the crude, unmatched, and unweighted cohort; the IPTW cohort; and the PSM cohort. Balance between covariates was assessed using absolute standardized mean differences; differences of 10% or less were considered to indicate an adequately balanced outcome. A *P* value of <0.05 was considered statistically significant. All analyses were performed using R (version 4.1.2; R Foundation for Statistical Computing, Vienna, Austria; https://www.r-project.org/).

## RESULTS

### Baseline characteristics

A total of 195 patients were screened, of whom 86 met the exclusion criteria and were excluded. Thus, a total of 109 patients were included in this study, of whom 69 received TACE-A and 40 received TACE-AP. PD-1 inhibitors were sintilimab (Innovent Pharmaceutical, Suzhou, China) in 14 cases (35%), camrelizumab (Hengrui Pharmaceutical, Lianyungang, China) in 11 cases (27.5%), tislelizumab (BeiGene Pharmaceutical, Shanghai, China) in 10 cases (25%), and pembrolizumab (Merck, Kenilworth, NJ) in 5 cases (12.5%). The baseline data of the 2 groups of patients showed differences, and the Child-Pugh scores of the 2 groups did not reach a sufficient balance (*P* < 0.05). After PSM and robust IPTW analyses, the baseline data of the 2 groups of patients reached a balance (Table 1).

**Table 1.** Baseline characteristics of the 2 groups before and after PSM and sIPTW analyses

Variable	Crude cohort					PSM cohort				Stabilized IPTW cohort			
	Grading	TACE-A (n = 69)	TACE-AP (n = 40)	*P* value	SMD	TACE-A (n = 28)	TACE-AP (n = 28)	*P* value	SMD	TACE-A (67.8)	TACE-AP (n = 40.2)	*P* value	SMD
Sex	Male	61 (88.4)	36 (90.0)	1.000	0.051	26 (92.9)	25 (89.3)	1.000	0.125	60.3 (88.9)	36.6 (90.9)	0.747	0.067
	Female	8 (11.6)	4 (10.0)			2 (7.1)	3 (10.7)			7.5 (11.1)	3.6 (9.1)		
Age	≤60	47 (68.1)	29 (72.5)	0.792	0.096	19 (67.9)	20 (71.4)	1.000	0.078	45.8 (67.6)	29.0 (72.0)	0.673	0.097
	>60	22 (31.9)	11 (27.5)			9 (32.1)	8 (28.6)			22.0 (32.4)	11.3 (28.0)		
Child-Pugh classification	A	54 (78.3)	38 (95.0)	0.041	0.507	26 (92.9)	26 (92.9)	1.000	<0.001	57.0 (84.1)	32.3 (80.2)	0.743	0.102
	B	15 (21.7)	2 (5.0)			2 (7.1)	2 (7.1)			10.8 (15.9)	8.0 (19.8)		
PVTT	TypeⅠ	46 (66.7)	27 (67.5)	0.694	0.171	21 (75.0)	18 (64.3)	0.540	0.300	44.7 (65.9)	25.3 (62.8)	0.849	0.130
	TypeⅡ	14 (20.3)	6 (15.0)			4 (14.3)	4 (14.3)			12.7 (18.7)	9.6 (23.9)		
	TypeⅢ	9 (13.0)	7 (17.5)			3 (10.7)	6 (21.4)			10.4 (15.3)	5.4 (13.4)		
Metastasis	None	42 (60.9)	19 (47.5)	0.248	0.271	14 (50.0)	15 (53.6)	1.000	0.072	36.6 (54.0)	21.6 (53.7)	0.982	0.005
	Have	27 (39.1)	21 (52.5)			14 (50.0)	13 (46.4)			31.2 (46.0)	18.6 (46.3)		
AFP (ng/mL)	<400	34 (49.3)	25 (62.5)	0.256	0.269	17 (60.7)	16 (57.1)	1.000	0.073	37.2 (54.9)	22.3 (55.4)	0.970	0.009
	≥400	35 (50.7)	15 (37.5)			11 (39.3)	12 (42.9)			30.5 (45.1)	17.9 (44.6)		
HBV	None	24 (34.8)	16 (40.0)	0.735	0.108	11 (39.3)	11 (39.3)	1.000	<0.001	25.2 (37.2)	14.0 (34.9)	0.836	0.049
	Have	45 (65.2)	24 (60.0)			17 (60.7)	17 (60.7)			42.6 (62.8)	26.2 (65.1)		
ECOG score	0	15 (21.7)	10 (25.0)	0.878	0.077	7 (25.0)	7 (25.0)	1.000	<0.001	16.5 (24.4)	9.0 (22.3)	0.823	0.051
	1	54 (78.3)	30 (75.0)			21 (75.0)	21 (75.0)			51.2 (75.6)	31.3 (77.7)		
TBIL (g/L)		23.32 (10.11)	22.96 (9.27)	0.850	0.038	22.07 (8.45)	22.88 (10.14)	0.747	0.087	23.01 (9.74)	24.50 (11.20)	0.658	0.141
ALB (μmol/L)		35.91 (5.13)	37.47 (5.15)	0.129	0.304	36.05 (4.94)	36.83 (5.56)	0.582	0.148	36.41 (5.09)	35.55 (6.36)	0.584	0.151
ALT (U/L)		49.90 (82.23)	36.92 (15.35)	0.326	0.219	37.57 (18.61)	38.79 (16.00)	0.794	0.070	44.00 (67.24)	34.71 (14.91)	0.202	0.191
Cr (μmol/L)		56.09 (11.34)	60.37 (16.21)	0.109	0.306	55.64 (9.72)	56.00 (8.68)	0.884	0.039	56.65 (11.17)	57.96 (12.20)	0.543	0.112
No. of liver tumors	1	6 (8.7)	7 (17.5)	0.393	0.263	3 (10.7)	3 (10.7)	0.850	0.153	6.3 (9.3)	3.9 (9.8)	0.905	0.091
	2	38 (55.1)	20 (50.0)			15 (53.6)	13 (46.4)			35.4 (52.3)	19.2 (47.8)		
	≥3	25 (36.2)	13 (32.5)			10 (35.7)	12 (42.9)			26.0 (38.4)	17.1 (42.4)		
Maximum tumor diameter (mm)		72.31 (37.51)	73.16 (35.45)	0.907	0.023	75.12 (34.33)	69.46 (33.52)	0.535	0.167	71.82 (36.64)	70.98 (31.36)	0.898	0.024
No. of TACE		1.88 (1.11)	1.90 (1.19)	0.944	0.014	1.64 (0.78)	1.86 (1.27)	0.450	0.204	1.84 (1.08)	1.83 (1.11)	0.974	0.007

ALB, albumin; ALT, alanine aminotransferase; AFP, alpha-fetoprotein; Cr, creatinine; ECOG, Eastern Cooperative Oncology Group; HBV, hepatitis B virus; PSM, propensity score matching; PVTT, portal vein tumor thrombus; sIPTW, stabilized inverse probability of treatment weighting; SMD, standardized mean differences; TACE, transarterial chemoembolization; TACE-A, TACE combined with apatinib; TACE-AP, TACE combined with apatinib and PD-1 inhibitors; TBIL, total bilirubin.

### Tumor response

In the crude cohort, the ORRs for the TACE-A and TACE-AP groups were 28.9% and 57.5%, respectively, and the difference between the 2 groups was statistically significant (*P* < 0.05) (Table 2). After PSM and sIPTW analyses, the ORRs for the TACE-AP group were 53.6% and 52.5%, respectively, which were significantly higher than 17.9% and 28.6% of the TACE-A group (*P* < 0.05).

**Table 2.** Objective tumor response rates before and after PSM and sIPTW analyses

Tumor response	Crude cohort		*P* value	PSM		*P* value	sIPTW		*P* value
	TACE-A (n = 69)	TACE-AP (n = 40)		TACE-A (n = 28)	TACE-AP (n = 28)		TACE-A (n = 67.8)	TACE-AP (n = 40.2)	
CR	2	3		0	1		1.8	3.9	
PR	18	20		5	14		17.6	17.2	
SD	29	8		16	8		29	11.8	
PD	20	9		7	5		19.4	7.3	
ORR (CR + PR)	29.0%	57.5%	0.003	17.9%	53.6%	0.005	28.6%	52.5%	0.013

CR, complete response; ORR, objective response rate; PD, progressive disease; PR, partial response; PSM, propensity score matching; sIPTW, stabilized inverse probability of treatment weighting; SD, stable disease; TACE, transarterial chemoembolization; TACE-A, TACE combined with apatinib; TACE-AP, TACE combined with apatinib and PD-1 inhibitors.

### Survival analysis

**Figure 2.**
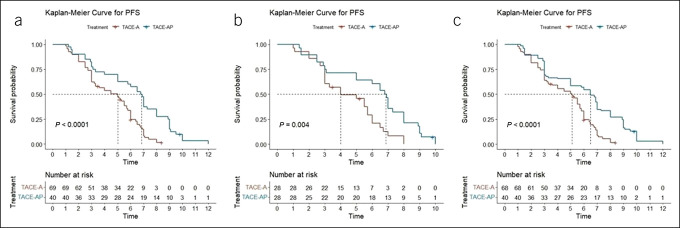
Kaplan-Meier analyses of PFS according to the TACE-A group and TACE-AP group in the crude cohort (**a**), PSM cohort (**b**), and sIPTW cohort (**c**). PFS, progression-free survival; PSM, propensity score matching; sIPTW, stabilized inverse probability of treatment weighting; TACE, transarterial chemoembolization; TACE-A, TACE combined with apatinib; TACE-AP, TACE combined with apatinib and PD-1 inhibitors.

As of the last follow-up (June 2022), in the crude cohort, the mPFS was 5.0 months (95% confidence interval [CI] 3.3–5.7) and 6.9 months (95% CI 5.0–8.0) in the TACE-A and TACE-AP groups (*P* < 0.001) (Figure 2a). After PSM analysis, the mPFS of the TACE-A and TACE-AP groups were 4.0 months (95% CI 3.0–6.0) and 6.9 months (95% CI 5.0–8.0), respectively (*P* = 0.004) (Figure 2b). After sTPTW analysis, the mPFS of the TACE-A and TACE-AP groups were 5.1 months (95% CI 3.3–5.8) and 6.5 months (95% CI 3.1–8.9), respectively (*P* < 0.001) (Figure 2c).

**Figure 3.**
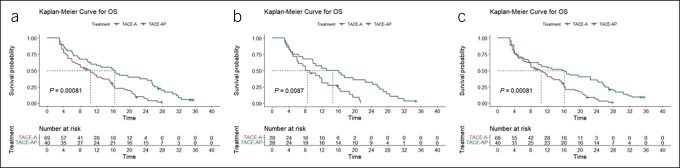
Kaplan-Meier analyses of OS according to the TACE-A group and TACE-AP group in the crude cohort (**a**), PSM cohort (**b**), and sIPTW cohort (**c**). OS, overall survival; PSM, propensity score matching; sIPTW, stabilized inverse probability of treatment weighting; TACE, transarterial chemoembolization; TACE-A, TACE combined with apatinib; TACE-AP, TACE combined with apatinib and PD-1 inhibitors.

In the crude cohort, the median OS was 10.5 months (95% CI 7.4–15.0) in the TACE-A group and 16.4 months (95% CI 9.9–25.5) in the TACE-AP group (*P* < 0.001) (Figure 3a). After PSM analysis, the median OS values of the TACE-A and TACE-AP groups were 8.5 months (95% CI 6.0–16.0) and 14.6 months (95% CI 9.8–25.5), respectively (*P* = 0.0087) (Figure 3b). After sTPTW analysis, the median OS values of the TACE-A and TACE-AP groups were 10.5 months (95% CI 8.0–15.0) and 16.1 months (95% CI 6.4–26.0), respectively (*P* < 0.001) (Figure 3c).

### OS-related prognostic factors

Univariate and multivariate Cox analyses were used to identify prognostic factors affecting OS (Table 3). In crude cohort and sIPTW cohort, univariate analysis showed that the prognostic factors affecting OS were tumor number, PVTT type, treatment modality, and alpha-fetoprotein (AFP) (*P* < 0.05). Multivariate Cox analysis showed that PVTT type (*P* < 0.001), AFP ≥400 ng/mL, and multiple tumors were independent risk factors of OS (*P* < 0.05), while TACE-AP treatment was an independent protective factor of OS (*P* < 0.05). However, in the PSM cohort, univariate and multivariate analyses showed that only PVTT type, treatment modality, and AFP were prognostic factors of OS (*P* < 0.05).

**Table 3.** Analyses of prognostic factors for survival before and after PSM and sIPTW analyses

Variable	Crude cohort				PSM				sIPTW			
	Univariate analysis		Multivariate analysis		Univariate analysis		Multivariate analysis		Univariate analysis		Multivariate analysis	
	HR (95% CI)	*P* value	HR (95% CI)	*P* value	HR (95% CI)	*P* value	HR (95% CI)	*P* value	HR (95% CI)	*P* value	HR (95% CI)	*P* value
Age	1.03 (0.67–1.58)	0.899			0.83 (0.46–1.51)	0.547			0.95 (0.61–1.50)	0.841		
Sex	0.87 (0.47–1.59)	0.641			0.95 (0.37–2.42)	0.917			0.83 (0.50–1.40)	0.492		
Child-Pugh classification = B	0.98 (0.54–1.75)	0.936			0.66 (0.2–2.22)	0.505			0.91 (0.55–1.48)	0.620		
Metastasis	0.94 (0.63–1.4)	0.768			0.98 (0.57–1.7)	0.946			0.98 (0.57–1.7)	0.963		
PVTT type II	2.73 (1.6–4.68)	0.000	2.49 (1.41–4.39)	0.001	2.72 (1.14–6.5)	0.025	2.91 (1.22–6.98)	0.017	2.97 (1.64–5.37)	0.000	2.81 (1.67–4.82)	0.000
PVTT type III	7.18 (3.9–13.2)	0.000	7.97 (3.96–16.03)	0.000	5.5 (2.46–12.31)	0.000	6.51 (2.47–17.14)	0.001	9.21 (4.41–19.24)	0.000	8.88 (3.91–20.18)	0.000
AFP ≥400 ng/mL	2.91 (1.91–4.45)	0.000	1.69 (1.07–2.68)	0.025	2.61 (1.47–4.64)	0.001	1.93 (1.00–3.73)	0.049	2.84 (1.74–4.65)	0.000	1.77 (1.06–2.96)	0.028
HBV	1.07 (0.71–1.61)	0.758			1.31 (0.74–2.33)	0.358			1.30 (0.77–2.19)	0.334		
ECOG = 1	0.75 (0.47–1.2)	0.231			0.79 (0.42–1.5)	0.473			0.84 (0.53–134)	0.461		
TBIL	1.01 (0.99–1.03)	0.344			1.02 (0.99–1.05)	0.118			1.02 (0.99–1.04)	0.146		
ALB	1.01 (0.97–1.04)	0.682			1.01 (0.97–1.06)	0.556			1.03 (0.98–1.08)	0.222		
ALT	1 (1–1)	0.810			1.01 (0.99–1.03)	0.159			0.99 (0.99–1.00)	0.864		
Cr	1 (0.98–1.01)	0.815			1 (0.97–1.03)	0.801			0.99 (0.98–1.01)	0.945		
Size	1 (1–1.01)	0.410			1 (0.99–1.01)	0.624			1.00 (0.99–1.00)	0.340		
Tumor number = 2	2.2 (1.13–4.29)	0.021	1.83 (0.92–3.64)	0.084	1.56 (0.63–3.84)	0.337			1.54 (0.85–2.78)	0.158	1.38 (0.75–2.55)	0.298
Tumor number ≥3	2.98 (1.48–6.01)	0.002	2.38 (1.15–4.91)	0.019	1.98 (0.79–4.96)	0.145			2.41 (1.38–4.18)	0.002	2.07 (1.17–3.71)	0.014
TACE	1.09 (0.94–1.28)	0.261			1.03 (0.82–1.29)	0.805			1.11 (0.96–1.28)	0.176		
Treatment	0.47 (0.3–0.73)	0.001	0.42 (0.26–0.68)	0.000	0.44 (0.24–0.82)	0.010	0.29 (0.15–0.57)	0.000	0.48 (0.27–0.88)	0.017	0.43 (0.24–0.76)	0.004

ALB, albumin; ALT, alanine aminotransferase; AFP, alpha-fetoprotein; CI, confidence interval; Cr, creatinine; ECOG, Eastern Cooperative Oncology Group; HR, hazard ratio; HBV, hepatitis B virus; PSM, propensity score matching; PVTT, portal vein tumor thrombus; sIPTW, stabilized inverse probability of treatment weighting; SMD, standardized mean differences; TACE, transarterial chemoembolization; TACE-A, TACE combined with apatinib; TACE-AP, TACE combined with apatinib and PD-1 inhibitors; TBIL, total bilirubin.

### Safety

Treatment-related AEs were observed in 101 (92.6%) of the 109 patients in this study and that of grade ≥3 AEs were 15 (13.8%) patients, and there were no treatment-related deaths. The most common treatment-related AEs in the 2 groups were fever, pain, nausea and vomiting, and fatigue, which were considered as postembolization syndrome after TACE treatment. The other common ones were hypertension, hand-foot syndrome, and loss of appetite. There was no significant difference in the incidence of treatment-related AEs between the 2 groups (*P* > 0.050). After symptomatic treatment and dose reduction or discontinuation, all treatment-related AEs were alleviated or eliminated. In the TACE-AP group, 1 patient (2.5%) developed reactive cutaneous capillary endothelial proliferation, 5 patients (12.5%) experienced hypothyroidism, and 2 experienced (2.5%) hyperthyroidism, and 1 patient (2.5%) experienced immune-related pneumonia, but the grades were all 1–2, which were relieved after symptomatic treatment (Table 4).

**Table 4.** Treatment-related adverse events in the 2 groups

Adverse events	TACE-A (n = 69)		TACE-AP (n = 40)	
	Any grades, n (%)	≥3 grade, n (%)	Any grades, n (%)	≥3 grade, n (%)
Fever	32 (46.4)	2 (2.9)	17 (42.5)	0
Pain	28 (40.6)	6 (4.3)	19 (47.5)	1 (2.5)
Nausea and vomiting	21 (30.4)	0	13 (32.5)	0
Hand–foot skin reactions	31 (44.9)	2 (2.9)	16 (40.0)	2 (5.0)
Hypertension	20 (30.0)	1 (1.4)	14 (35.0)	1 (2.5)
Proteinuria	2 (3.0)	0	1 (2.5)	0
Fatigue	5 (6.67)	0	3 (7.5)	0
Hoarseness	5 (7.2)	0	1 (2.5)	0
Oral ulcer	4 (5.8)	0	2 (5.0)	0
Rash	3 (4.3)	0	0 (0.0)	0
Gastrointestinal hemorrhage	4 (5.8)	0	2 (5.0)	0
Gastrointestinal reaction	10 (14.5)	0	7 (17.5)	0
RCCEP	0	0	1 (2.5)	0
Hypothyroidism	0	0	5 (12.5)	0
Hyperthyroidism	0	0	2 (5.0)	0
Pneumonia	0	0	1 (2.5)	0

RCCEP, reactive cutaneous capillary endothelial proliferation; TACE, transarterial chemoembolization; TACE-A, TACE combined with apatinib; TACE-AP, TACE combined with apatinib and PD-1 inhibitors.

## DISCUSSION

This study compared the efficacy and safety of TACE-A and TACE-AP in the treatment of HCC with PVTT, in which the median OS of TACE-AP was 16.1 months, which is the first report to our knowledge.

The American Association for the Study of Liver Diseases and the European Association for the Study of Liver Diseases currently recommend sorafenib as the first-line treatment for patients with HCC with PVTT (11,12). Sorafenib targets multiple tyrosine kinases, including RAF, VEGFR, and PDGFR, while apatinib selectively inhibits VEGFR-2, and apatinib binding affinity to VEGFR-2 is 10 times that of sorafenib; therefore, the antiangiogenesis effect of apatinib is not weaker than that of sorafenib (13,14). Previous studies have demonstrated synergistic effects of TACE in combination with TKIs. TACE causes tumor tissue hypoxia and induces increased secretion of VEGF, which in its turn induces tumor neovascularization and promotes the proliferation of residual tumor cells, leading to tumor recurrence and metastasis. Furthermore, the application of TKIs can inhibit neovascularization and thus tumor cell proliferation. Mechanistically, the combination of TACE and TKIs can reduce the recurrence and metastasis of tumors (15). In a study comparing the efficacy and safety of TACE combined with sorafenib or apatinib in the treatment of HCC with PVTT, the median OS of the sorafenib group and the apatinib group were 11.0 months and 10.0 months, respectively (16 ). In another study of TACE combined with apatinib in the treatment of tumor thrombus in the first and higher branches of the portal vein, the median OS was 12.2 months (17). The median OS in the TACE-A group in this study was 10.5 months, which was similar to that in previous studies. These studies suggest that TACE combined with apatinib is effective in patients with HCC with PVTT.

Recent studies have shown that antiangiogenic drugs combined with immune checkpoint inhibitors may result in greater survival benefits for the treatment of liver cancer with portal tumor thrombus (18). TKIs with antiangiogenic properties are the standard first-line therapy for advanced HCC. A growing number of studies confirm that TKIs (sorafenib, lenvatinib, regorafenib, cabozantinib, etc.) have immunomodulatory effects on the tumor microenvironment (19–21). These immunomodulating effects include the promotion of dendritic cell maturation, upregulation of T-cell trafficking and function, and reversal of immunosuppression cell expression caused by tissue hypoxia. Therefore, modulation of the tumor microenvironment by targeted TKIs often enhances the therapeutic effect of PD-1 inhibitors (22). Synergy of TKIs plus PD-1 inhibitors has been demonstrated in many cancers (23). TACE combined with targeted therapy and immunotherapy is more effective in improving survival in patients with advanced HCC (24–27). In general, there are few reports on targeted therapy combined with immunotherapy for patients with HCC with PVTT, and there is no report on TACE combined with TKIs and PD-1 inhibitors therapy. A number of recent studies have explored TACE combined with TKIs and PD-1 inhibitors in the treatment of advanced HCC, and the median OS was greater than 20 months, and the efficacy was significantly higher than that observed in our study (the median OS in the TACE-AP group was 16 months) (28,29); the possible reason is that there is a large difference in baseline characteristics. All the patients included in our study are patients with HCC with PVTT. Previous studies have confirmed that PVTT is an important factor affecting survival, while the proportion of patients included in the abovementioned studies with PVTT is less than 50%. Another possible reason is that the tumor burden of our patients is large, the average diameter of the largest tumor is >70 mm, and the proportion of multiple tumors is more than 60%, which may also greatly shorten the survival time of patients.

Multivariate analysis in this study showed that the main factors associated with OS were PVTT type, tumor number, AFP, and treatment, which was the same as those observed in the previous study. However, the results of multifactor analysis after PSM show that the number of tumors has no impact on OS. The possible reason is that PSM matches only 28 pairs of patients, and the data are lost a lot (data of 53 patients were lost). The crude cohort is the same as the sIPTW cohort, which indicates that the application of different statistical methods may lead to deviation of conclusions. Therefore, when selecting statistical methods, we should combine the clinical situation to reach a more reasonable and reliable conclusion.

Liver function has been previously reported to be associated with patient prognosis; however, it was not a prognostic factor in this study. This could be because all our patients had good liver function (Child-Pugh A or B ≤7), because deteriorated liver function is currently a potential contraindication for the systemic treatment of HCC (30). Notably, Granito et al (31) found that a postprocedure increase of transaminases (aspartate transaminase increase ≥46%, alanine transaminase increase ≥52%) compared with baseline values was shown to be a reliable predictor of response to TACE. The correlation between changes in biochemical parameters and tumor response after TACE is an exciting future area of investigation. Although preoperative aminotransferases were not a prognostic factor in our study, unfortunately, biochemical indicators after TACE were not collected. Therefore, the correlation between changes in transaminase levels before and after TACE and tumor response could not be analyzed.

The incidence of treatment-related AEs in this study was basically consistent with those in previous studies (32–34). TACE-related AEs are mainly postembolization syndrome, including fever, pain, nausea, and vomiting, and AEs related to apatinib and PD-1 inhibitors mainly include hand–foot skin reaction, hypertension, fatigue, oral ulcers, proteinuria and rash, thyroid dysfunction, myocarditis and pneumonitis; symptoms associated with these AEs resolved or resolved after symptomatic treatment or temporary discontinuation of the drug. The incidence of TRAEs was basically the same between the TACE-A and TACE-AP groups, and the difference was not statistically significant (*P* > 0.05), which indicated that the combination of PD-1 inhibitors did not increase TACE and apatinib adverse reactions.

Our study has certain limitations. First, this is a multicenter retrospective study, and although we have balanced the baseline characteristics of the 2 groups by using PSM and IPTW analyses, the potential patient selection bias cannot be completely avoided. Second, a variety of different PD-1 inhibitors were included in this study, and the consistency of efficacy may not be guaranteed. In addition, after the first TACE treatment, some patients did not receive apatinib plus PD-1 inhibitor regularly because of economic reasons. If they had received the combination therapy regularly, they could have obtained higher clinical benefits. Therefore, the conclusions of this study need to be further confirmed by prospective, multicenter, randomized controlled trials.

In conclusion, for patients with HCC PVTT, compared with TACE-A, TACE-AP significantly improved patient survival and ORR, while treatment-related AEs were safe and controllable.

## CONFLICTS OF INTEREST

**Guarantor of the article:**

**Specific author contributions:** H.-L.L., H.-T.H., and S.-J.X.: conception and design the study. W.-L.X., G.-S.C., Z.L., and W.-J.F.: provision of study materials or patients. W.-L.X., Y.G., X.-H.Z., and S.-J.X.: collection and assembly of data. X.-H.Z. and Y.G.: data analysis and interpretation. W.-L.X.: manuscript writing. H.-L.L., H.-T.H., and S.-J.X.: manuscript reviewing. All authors: final approval of manuscript.

**Financial support:** This work was supported by The National Natural Science Foundation (82002596), Henan Province Natural Science Foundation (212300410403), Medical Science and Technology Research Project of Henan Province (No. LHGJ20190633), Science and Technology Department of Henan Province (No. 212102310162), Beijing Health Alliance Charitable Foundation (HN-20201017-001), and the Technology Major Project of the Ministry of Science and Technology of China (2018ZX10303502).

**Potential competing interests:** None to report.

## Notes

Study Highlights WHAT IS KNOWN

✓ The treatment of HCC with PVTT is difficult and inconclusive.
✓ The effectiveness of the combination of TACE and apatinib plus PD-1 inhibitors has not been fully evaluated.

WHAT'S NEW HERE

✓ We analyzed the difference in prognosis between TACE-AP and TACE-A for PVTT in patients with HCC.
✓ PSM and sIPTW are important statistical methods for balancing covariates.

THE IMPACT OF CONVERSION

TACE-AP may be a more effective treatment option for PVTT in patients with HCC compared with TACE-A alone.
